# Electric LAMP: Virtual Loop-Mediated Isothermal AMPlification

**DOI:** 10.5402/2012/696758

**Published:** 2012-11-21

**Authors:** Nelson R. Salinas, Damon P. Little

**Affiliations:** ^1^Cullman Program for Molecular Systematics, The New York Botanical Garden, Bronx, NY 10458, USA; ^2^The Graduate Center, The City University of New York, New York, NY 10016, USA

## Abstract

We present eLAMP, a PERL script, with Tk graphical interface, that electronically simulates Loop-mediated AMPlification (LAMP) allowing users to efficiently test putative LAMP primers on a set of target sequences. eLAMP can match primers to templates using either exact (via builtin PERL regular expressions) or approximate matching (via the tre-agrep library). Performance was tested on 40 whole genome sequences of *Staphylococcus*. eLAMP correctly predicted that the two tested primer sets would amplify from *S. aureus* genomes and not amplify from other *Staphylococcus* species. Open source (GNU Public License) PERL scripts are available for download from the New York Botanical Garden's website.

## 1. Introduction

Several highly efficient methods of DNA amplification have been proposed. Although the Polymerase Chain Reaction (PCR; [1, 2]) is the most widely used method, newer more sensitive techniques are favored for some applications—particularly diagnostic testing. Loop–mediated isothermal AMPlification (LAMP; [3]) is perhaps the most promising of these new methods [4]. LAMP can quickly generate large quantities of amplicon from low abundance template without temperature cycling—thereby lowering the cost and complexity of necessary laboratory equipment. In addition, LAMP amplicons, unlike PCR amplicons, can be directly detected via a colorimetric chemical reaction [5, 6]. 

Unlike PCR which requires one pair of primers, LAMP requires a minimum of two nested primer pairs. Generally, primers should be 15–25 bases long with 40–60% GC content (annealing 55–65°C). The amplicon defined by the outer primers should be ≤280 base pairs and the spacing between inner and outer primers including the outer primers ≥40 base pairs [5, 7]. Primers are synthesized such that the reverse complements of the inner primers are connected to the outer primers by a linker (usually five thymine residues). Thus, interconnected loops are produced during amplification [3]. Optionally, additional sets of primers can be used to increase amplification efficiency [3, 7–9].

The quality of LAMP primer/template match (i.e., the percent mismatched bases and the distribution of mismatches) necessary for efficient amplification has not been studied, but there are studies of PCR primer/template match quality that suggest an exact 3′ match of 2-3(4) bases is required for *Taq* polymerase to extend the primer along the template [10–14] and mismatches outside of the 3′ end of the primer have little effect on amplification efficiency [10, 14]. Although PCR and LAMP have many similarities, different polymerases and extension temperatures are typically used. It is not known to what degree the conclusions from these studies on PCR can be applied to LAMP.

Although LAMP is increasingly used for diagnostic testing (e.g., [15–19]), electronic tools for LAMP are limited to primer design [20, 21]. One of the available tools, LAVA [21], can design LAMP primers from aligned sequences and thus could be used to design either universal or selectively discriminatory primers. Unfortunately LAVA cannot be used to predict the activity of existing primer sets—useful when one wishes to determine if a newly discovered sequence variant can be amplified with existing primers. Electronic testing of primers allows researchers to quickly and inexpensively determine if newly designed primers will work with known sequence variants. This is particularly useful when meaningful consensus sequences cannot be used in primer design (e.g., difficult to align regions). Because two, or more, nested primer sets are used, LAMP cannot be directly modeled with conventional PCR simulation software (e.g., re-PCR [22]). Here we present eLAMP, a PERL script that simulates LAMP and outputs an estimate of amplification success given target sequence(s) and primers.

## 2. Algorithm 

By default, input template(s) are evaluated only in the given orientation, but a user option allows evaluation of both orientations (resulting in roughly double the analysis time). Input primers are checked for compliance with standard LAMP design parameters—this can be overridden by the user. Each user provided template is checked against all inner primers. Results are stored and the process is repeated separately with outer primers. The matching algorithm is the same for all primer pairs.

1. Each pair is divided into forward (left) and reverse (right) primers.
2. The reverse primer is converted to its reverse-complement.
3. Exact matching. If there are exact matches between the forward primer and the template, the reverse primer is used for additional exact matching. Otherwise approximate matching is initiated (step 4).
  - For each forward exact match, the reverse primer is checked against the template within an expected range (by default 1–51 bp for inner, 81–280 for outer primers, and ≥25 bp between adjacent inner and outer primers, other values may be specified by the user; [3, 5, 7]). If a match is found, both primer-binding positions are stored. If no exact match is found for the reverse primer, approximate matching is initiated (step 4).
4. Approximate matching. This procedure is triggered only if there are no exact matches, for both primers, and approximate matching parameters have been set. The agrep algorithm [23] is used for approximate matching. The user specifies the number of exact 3′ matches (1–3) and the percent of matching bases for the remainder of the primer.
  - Approximate matches between template and the forward primer are stored.
  - If there are any forward matches, reverse primer approximate matches are identified and stored.
  - Pairs of forward and reverse positions that would produce an amplicon within an expected range (see step 3a) are saved.

For each template and primer set, if the predicted annealing positions of all constituent primer pairs are nested and appropriately spaced (see step 3a), eLAMP predicts successful amplification.

## 3. Implementation

**Figure 1:**
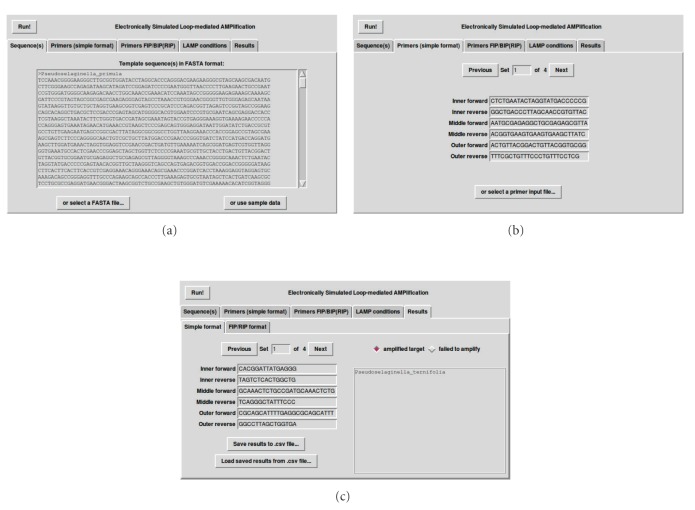
User interface for eLAMP. Once the user has (a) input template and (b) specified primer sequences, (c) amplification success/failure can be estimated.

The algorithm has been implemented as a GNU Public License (GPL) PERL script (http://www.nybg.org/files/scientists/dlittle/eLAMP.html). Tre (http://laurikari.net/tre/) provides agrep functionality. A graphic user interface—for Mac OS X, LINUX, and Windows—is provided by PERL/Tk (Figure 1). 

Users of eLAMP specify a  .fasta file with template sequence(s) and a comma-separated value (.csv) file of primers. Each cell of the  .csv file corresponds to a primer and each line to a primer set. Primer pairs are ordered from the innermost to the outermost. Within each primer pair, the forward (left) primer should be followed by the reverse (right). A line of column headers is optional. The alternative FIB/BIP primer format can be used, but linkers must be delimited by hyphens (e.g., “–TTTTT–”). Output is a  .csv file: the first row indicates the primer set(s) and the first column the template(s). Success or failure in each amplification is coded as 1 or 0, respectively.

## 4. Empirical Example

Electronic amplification of the diagnostic *gltA/gltB* region was attempted from all 40 complete *Staphylococcus* genomes deposited in GenBank (Table 1; median = 2,813,126 base pairs, IQR = 180,199 base pairs). Two similar and overlapping sets of primers designed to only amplify from *S. aureus* were used ([21]; LAVA F1: 3′-GGA ATA GTT TGT AAG ACA CCT GC CA-5′, LAVA R1: 3′-CAA AAA CAA AGC GAA CTG CCA AT-5′, LAVA F2: 3′-ACC AAC ACC AAA AAT CGG T-5′, LAVA R2: 3′-TGG CAT TAT TAC TTG CCA TCA-5′, LAVA F3: 3′-GCT ACA ATT GCA GGC GTT T-5′, LAVA R3: 3′-TTG ATG TCG AAA ACA CTG GAA-5′; PrimerExplorer F1: 3′-TGT TGG AAT AGT TTG TAA GAC ACC T-5′, PrimerExplorer R1: 3′-CAA AAA CAA AGC GAA CTG CCA ATA-5′, PrimerExplorer F2: 3′-TTA CCA ACA CCA AAA ATC GG-5′, PrimerExplorer R2: 3′-GCA TTA TTA CTT GCC ATC ATT G-5′, PrimerExplorer F3: 3′-GCT ACA ATT GCA GGC GTT-5′, PrimerExplorer R3: 3′-TGT CGA AAA CAC TGG AAC AT-5′).

Reported times are the median of five sequential single-threaded executions on an Intel Pentium D 950 (3.4 GHz) with 4 GB of 533 MHz RAM running 64-bit Ubuntu 12.04.1 LTS. All files were placed on a 256 MB RAM disk (a tmpfs volume) prior to analysis. eLAMP was instructed to evaluate the genome sequence in both possible orientations, to perform exact primer matching, and to perform approximate matching (an exact match of 3′ ultimate, penultimate, and antepenultimate bases and 75% similarity for the remaining bases).

The two primer sets behaved similarly (Table 1): the exact matching procedure predicted, depending on the primer set, a LAMP amplicon for 25 or 26 of the 31 *S. aureus* genomes. Approximate matching resulted in a predicted LAMP amplicon for 29 of the 31 *S. aureus* genomes. Although the total predicted by approximate matching was the same for both primer sets, predictions for CP003166.1 and CP003194.1 varied by primer set. No matter the primer set or the matching procedure used, none of the nine remaining genomes, representing six other *Staphylococcus* species, were predicted to produce an amplicon. 

The *S. aureus* genomes not predicted to produce an amplicon have various mismatches: a single mismatch within the F1 primers of both primer sets (CP001996.1, FR821779.1); a single mismatch within the F1 primers of both primer sets, different from the above mismatch (HE681097.1); a mismatch at the penultimate 3′ base of the LAVA F1 primer (CP003166.1); a single mismatch within the F2 primers of both primer sets (including the antepenultimate 3′ base of the PrimerExplorer primer) as well as a single mismatch within the LAVA R2 primer (CP003194.1); and single mismatches within the F3 and R1 primers of both primer sets as well as multiple mismatches within both primer sets for the F1 (including the ultimate 3′ base of the PrimerExplorer primer), R2 (including the penultimate 3′ base of the PrimerExplorer primer), and R3 (including the ultimate 3′ bases of both primer sets; FR821777.2). Given the large number of mismatches and their distribution, eLAMP does not predict amplification of the *S. aureus *sequence FR821777.2 with either exact or approximate matching. This sequence is from a highly divergent strain of *S. aureus* that could, arguably, be classified as a different species—*S. argenteus* [24]. 

The sequences of 14 *S. aureus* genomes were considered when the LAVA and PrimerExplorer primer sets were designed ([21]; AJ938182.1, AP009324.1, AP009351.1, BA000017.4, BA000018.3, BA000033.2, BX571856.1, BX571857.1, CP000046.1, CP000253.1, CP000255.1, CP000703.1, CP000730.1, and CP000736.1). These genomes were predicted to produce a LAMP amplicon for both primer sets under both matching procedures. The *S. aureus* genomes that were, under various circumstances, predicted to not produce any amplification were not consulted when primers were designed (CP001996.1, CP003166.1, CP003194.1, FR821777.2, FR821779.1, and HE681097.1). Given the assumptions of match quality, neither primer set is entirely diagnostic of *S. aureus—*either the primers must be used in combination or, ideally, new primers that use less variable sites should be designed.

eLAMP with exact primer matching required 10.556 seconds and approximate matching required 9 minutes 18.608 seconds.

## 5. Conclusions

eLAMP is a free PERL script that simulates loop–mediated isothermal amplification. It provides a fast and inexpensive test of LAMP primer suitability. The graphical interface is simple—allowing easy use by nonspecialists—while the command-line interface is suitable for use in pipelines. Results, presented either in a single  .csv file or in a GUI panel, are straightforward to interpret.

## Figures and Tables

**Table 1:** eLAMP amplification, using two primer sets, of the *gltA/gltB* region from 40 complete *Staphylococcus* genomes (0 = no amplification; 1 = amplification).

Species	GenBank accession	Exact matching		Approximate matching	
		LAVA	PrimerExplorer	LAVA	PrimerExplorer
*S. aureus *	AJ938182.1	1	1	1	1
*S. aureus *	AM990992.1	1	1	1	1
*S. aureus *	AP009324.1	1	1	1	1
*S. aureus *	AP009351.1	1	1	1	1
*S. aureus *	BA000017.4	1	1	1	1
*S. aureus *	BA000018.3	1	1	1	1
*S. aureus *	BA000033.2	1	1	1	1
*S. aureus *	BX571856.1	1	1	1	1
*S. aureus *	BX571857.1	1	1	1	1
*S. aureus *	CP000046.1	1	1	1	1
*S. aureus *	CP000253.1	1	1	1	1
*S. aureus *	CP000255.1	1	1	1	1
*S. aureus *	CP000703.1	1	1	1	1
*S. aureus *	CP000730.1	1	1	1	1
*S. aureus *	CP000736.1	1	1	1	1
*S. aureus *	CP001781.1	1	1	1	1
*S. aureus *	CP001844.2	1	1	1	1
*S. aureus *	CP001996.1	0	0	1	1
*S. aureus *	CP002110.1	1	1	1	1
*S. aureus *	CP002114.2	1	1	1	1
*S. aureus *	CP002120.1	1	1	1	1
*S. aureus *	CP002643.1	1	1	1	1
*S. aureus *	CP003033.1	1	1	1	1
*S. aureus *	CP003045.1	1	1	1	1
*S. aureus *	CP003166.1	0	1	0	1
*S. aureus *	CP003194.1	0	0	1	0
*S. aureus *	FN433596.1	1	1	1	1
*S. aureus *	FR714927.1	1	1	1	1
*S. aureus *	FR821777.2	0	0	0	0
*S. aureus *	FR821779.1	0	0	1	1
*S. aureus *	HE681097.1	0	0	1	1
*S. carnosus *	AM295250.1	0	0	0	0
*S. epidermidis *	AE015929.1	0	0	0	0
*S. epidermidis *	CP000029.1	0	0	0	0
*S. haemolyticus *	AP006716.1	0	0	0	0
*S. lugdunensis *	CP001837.1	0	0	0	0
*S. lugdunensis *	FR870271.1	0	0	0	0
*S. pseudintermedius *	CP002439.1	0	0	0	0
*S. pseudintermedius *	CP002478.1	0	0	0	0
*S. saprophyticus *	AP008934.1	0	0	0	0
